# Autophagy, a guardian against neurodegeneration

**DOI:** 10.1016/j.semcdb.2010.02.008

**Published:** 2010-09

**Authors:** Moisés García-Arencibia, Warren E. Hochfeld, Pearl P.C. Toh, David C. Rubinsztein

**Affiliations:** Department of Medical Genetics, University of Cambridge, Cambridge Institute for Medical Research, Addenbrooke's Hospital, Hills Road, Cambridge CB2 0XY, UK

**Keywords:** Autophagy, Alzheimer disease, Neurodegeneration, Huntington disease

## Abstract

Autophagy is an intracellular degradation process responsible for the clearance of most long-lived proteins and organelles. Cytoplasmic components are enclosed by double-membrane autophagosomes, which subsequently fuse with lysosomes for degradation. Autophagy dysfunction may contribute to the pathology of various neurodegenerative disorders, which manifest abnormal protein accumulation. As autophagy induction enhances the clearance of aggregate-prone intracytoplasmic proteins that cause neurodegeneration (like mutant huntingtin, tau and ataxin 3) and confers cytoprotective roles in cell and animal models, upregulating autophagy may be a tractable therapeutic strategy for diseases caused by such proteins. Here, we will review the molecular machinery of autophagy and its role in neurodegenerative diseases. Drugs and associated signalling pathways that may be targeted for pharmacological induction of autophagy will also be discussed.

## 1 The basics of autophagy

Autophagy is an intracellular catabolic system in which cytoplasmic components are delivered into lysosomes for degradation. Three different autophagic pathways are known: (1) macroautophagy, (2) microautophagy, and (3) chaperone-mediated autophagy (CMA). In macroautophagy, a double-membraned structure (called a phagophore) elongates engulfing a portion of cytoplasm, and then fuses to form a vesicle called an autophagosome. Autophagosomes fuse with lysosomes, thereby forming autolysosomes, where the cytosolic contents are degraded by lysosomal hydrolases. In microautophagy, a portion of cytoplasm is directly engulfed into lysosomes by invagination of the lysosomal membrane. CMA involves the selective transport of cytosolic proteins that contain a pentapeptide motif related to KFERQ across the lysosomal membrane via the chaperone hsc70 and the lysosomal membrane receptor LAMP-2A.

In this review, we will focus on mammalian macroautophagy, hereafter referred to as autophagy, and its roles in neurodegeneration. Basal autophagy plays an important role in cellular homeostasis, through the degradation of long-lived proteins, protein aggregates and organelles. In addition, autophagy can also be induced as a cellular reaction to various physiological and pathological situations, such as nutrient starvation, or pathogen infection. Thus, dysfunction in autophagy has been implicated in the pathogenesis of various diseases, like cancer, infectious diseases and neurodegenerative disorders [1].

## 2 Autophagy machinery

Our understanding of the molecular machinery of autophagy started with the identification of the autophagy-related (*ATG*) genes in *Saccharomyces cerevisiae*. To date, more than 30 different *ATG* genes have been identified in yeast [2], and many of them have mammalian orthologues.

### 2.1 Initiation of autophagosome formation

Autophagosome formation occurs at the phagophore-assembly-site(s) (PAS) [3]. The activity of Vps34, a class III phosphatidylinositol-3-kinase (PI3K), is necessary for the formation of new autophagosomes. Vps34 generates phosphatidylinositol-3-phosphate (PI3-P) at the PAS, which allows the recruitment of other Atg proteins. Vps34 is part of the autophagy-regulating macromolecular complex (PI3K complex), which contains Beclin 1/Atg6, Atg14/barkor and p150/Vps15, among other proteins [4]. The activity of Vps34 is enhanced by its binding to Beclin 1. Positive regulators of Beclin 1 function and autophagy include AMBRA1, UVRAG and Bif-1, whereas its negative regulators include the anti-apoptotic proteins Bcl-2 and Bcl-X_L_ [5]. The other protein complex involved in this stage of autophagosome formation is the ULK1/Atg1–Atg13–FIP200/Atg17–Atg101 complex [6]. This complex plays an important role in both the recruitment of Atg proteins and the subsequent autophagosome synthesis.

### 2.2 Elongation

Two ubiquitin-like conjugation systems are involved in the elongation and expansion of the phagophore membrane. In the first conjugation event, Atg12 is conjugated to Atg5 in a reaction that requires Atg7 (E1-like) and Atg10 (E2-like) [7]. The Atg12–Atg5 conjugate interacts non-covalently with Atg16L, which oligomerizes to form an 800-kDa complex [8], which is necessary for autophagosome formation.

The second ubiquitination-like reaction involves the conjugation of microtubule-associated protein 1 light chain 3 (MAP1-LC3/LC3/Atg8) to the lipid phosphatidylethanolamine (PE). LC3 is cleaved at its C terminus by Atg4 to form the cytosolic LC3-I, which is conjugated with PE through the action of Atg7 (E1-like) and Atg3 (E2-like) to generate LC3-II [9]. LC3-II is the most widely used marker to study autophagy, as it is the only known protein that specifically associates with autophagosomes and not with other vesicular structures. LC3-II is bound to both sides of the membrane, and it remains membrane bound even after fusion with lysosomes, after which LC3-II on the cytosolic face of autophagosomes can be recycled (to LC3-I) by Atg4 [10], while the LC3-II on the inner face of the membrane is degraded.

### 2.3 Maturation and fusion

Mammalian autophagosomes form randomly in the cytoplasm. They are then trafficked along microtubules in a dynein-dependent manner to lysosomes, which are clustered around the microtubule-organising center (MTOC) located near the nucleus [11]. The details of the autophagosome–lysosome fusion in mammalian autophagy are still unclear, although it is thought that the fusion step involves proteins such as ESCRT, SNAREs, Rab7, UVRAG, LAMP-2 and the class C Vps proteins [12,13].

## 3 Signalling pathways regulating mammalian autophagy

### 3.1 mTOR-dependent pathway

**Fig. 1:**
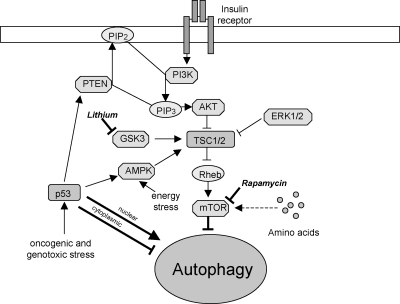
mTOR-dependent pathway, with drugs acting at distinct stages in this pathway enhancing autophagy.

The mammalian target of rapamycin (mTOR) kinase is a master negative regulator of autophagy [14] (Fig. 1). mTOR is a central sensor of energy status, growth factors and nutrient signals, and can be inhibited by drugs such as rapamycin [15]. Under nutrient-rich conditions, mTOR suppresses autophagy through direct interaction with the ULK1–Atg13–FIP200 complex and mediates phosphorylation-dependent inhibition of the kinase activity of Atg13 and ULK1. Under starvation conditions or rapamycin treatment, mTOR-mediated phosphorylation of Atg13 and ULK1 is inhibited. This leads to dephosphorylation-dependent activation of ULK1 and ULK1-mediated phosphorylations of Atg13, FIP200, and ULK1 itself, which trigger autophagy [6].

A major signalling cascade regulating mTOR activity is the PI3K pathway. The binding of growth factors or insulin to cell surface receptors activates the class 1a PI3K. Activated PI3K catalyzes the production of phosphatidylinositol-3,4,5-triphosphate (PIP_3_) at the plasma membrane, which increases the membrane recruitment of Akt/PKB and its activator PDK1, leading to the activation of Akt. The phosphorylation-dependent Akt activation results in the phosphorylation of a host of other proteins, including the tuberous sclerosis complex 1/2 (TSC1/TSC2). The TSC1/TSC2 complex integrates upstream signals from various kinases, including AKT and ERK1/2 [16]. Phosphorylation of TSC2 by these kinases leads to the disruption of the heterodimer with TSC1, resulting in loss of TSC1/TSC2 activity. Since TSC1/TSC2 acts as the GTPase-activating protein (GAP) for the Ras-family GTP binding protein, Rheb, which directly binds and activates mTOR [17], loss of TSC1/TSC2 activity results in mTOR activation.

mTOR can also act as a sensor of changes in cellular energy states via AMPK [18]. AMPK, activated when cells are energy-depleted as it senses changes in intracellular AMP/ATP ratios, directly phosphorylates TSC2, thereby providing the priming phosphorylation for subsequent phosphorylation of TSC2 by glycogen synthase kinase 3 (GSK3) to inhibit mTOR signalling [19].

The p53 tumor suppressor, encoded by a commonly mutated gene in human cancers, can positively and negatively regulate autophagy depending on its localization. Oncogenic or genotoxic stress stabilizes and activates nuclear p53. This can stimulate autophagy by activating AMPK or by upregulating phosphatase and tensin homologue (PTEN, a PIP_3_ 3′ phosphatase) and TSC1 which inhibits mTOR. Cytoplasmic p53, however, inhibits autophagy [20].

Recently, further insights have been provided into the mechanisms behind starvation-induced autophagy. Autophagy can be inhibited by the binding of the apoptosis-related proteins Bcl-2 or Bcl-X_L_ to Beclin 1. Starvation induces Jun N-terminal kinase 1 (Jnk1) activity, which phosphorylates Bcl-2, thereby disrupting the interaction between Beclin 1 and Bcl-2 to induce autophagy [21]. This mechanism might also account for the upregulation of autophagy after proteasome inhibition or ER stress, as one study shows that ER-stress-induced autophagy is Jnk1-dependent and that proteasome inhibition can induce ER stress [22].

### 3.2 mTOR-independent pathway

**Fig. 2:**
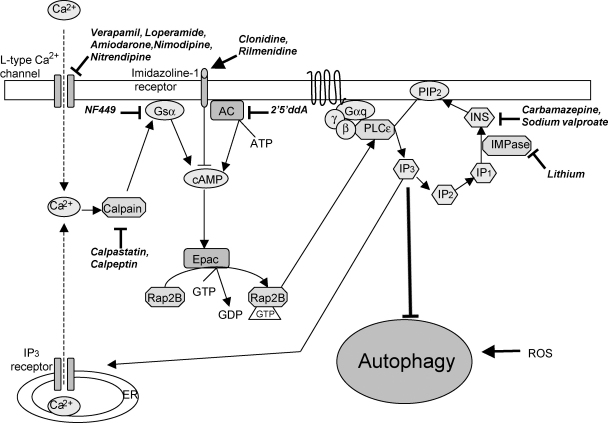
mTOR-independent pathway, with multiple drug targets acting at various places in this pathway that induce autophagy.

The first evidence for the existence of mTOR-independent regulation of mammalian autophagy comes from studies showing that autophagy is negatively regulated by intracellular inositol and inositol 1,4,5-trisphosphate (IP_3_) leves [23] (Fig. 2). Inhibition of inositol monophosphatase (IMPase) reduces free inositol and IP_3_ levels, which leads to an upregulation of autophagy. Autophagy is inhibited when intracellular cAMP levels are increased by adenylyl cyclase (AC). cAMP inhibits autophagy via Epac, which is a guanine nucleotide exchange factor. Epac then activates Rap2B, that subsequently activates phospholipase Cɛ (PLCɛ), resulting in the production of IP_3_, which mediates the release of Ca^2+^ from ER stores. Increased intracytosolic Ca^2+^ blocks autophagy by activating calpains (a family of Ca^2+^-dependent cysteine proteases), which mediate their effects on autophagy through Gsα, which is activated after calpain cleavage. This, in turn, increases AC activity to increase cAMP levels. This creates a potential cyclic pathway where calpain regulates autophagy through Gsα that signals via the cAMP–Epac–PLCɛ–IP_3_ pathway, which modulates calpain activity by influencing Ca^2+^ levels [24].

Autophagy is also modulated by reactive oxygen species (ROS). The generally deleterious effect of ROS on intracellular structures and their association with disease means they have traditionally been considered as harmful. However, ROS also have physiological roles in the cell, notably in signalling [25]. Starvation (a potent inducer of autophagy) increases levels of ROS in a PI3K-dependent manner, and treatment with antioxidants ameliorates the ability of starvation to induce autophagy [26]. One way in which ROS may be acting to regulate autophagy is by the modulation of the action of Atg4 on Atg8/LC3. The Atg4-mediated delipidation of LC3-II on the cytosolic surface of autolysosomes allows it to be recycled. Atg4 is inactive and unable to cleave Atg8 from membranes when in its oxidized state [27]. It is therefore possible that under oxidative conditions Atg4 is oxidized and inactive, which allows Atg8 to lipidate and thus initiates autophagy, while reduced Atg4 is active favoring Atg8 delipidation.

## 4 Neurological diseases caused by mutations that compromise autophagy

One of the common pathological features of most adult-onset human neurodegenerative diseases is the formation of intracytoplasmic aggregates within neurons and other cell types. This is seen in Alzheimer disease and in tauopathies (where tau accumulates in the cytoplasm), in Parkinson disease (where α-synuclein is the major component of the aggregates), and in polyglutamine expansion diseases like Huntington disease, where the mutant protein is the primary constituent of the aggregates. A common theme emerging in these studies is the role of autophagy in degrading disease-related, aggregate-prone, mutant, intracytoplasmic proteins such as tau, huntingtin and mutant α-synuclein [28]. Extensive data suggest that such aggregate-prone proteins mediate toxicity primarily via gain-of-function mechanisms associated with their propensity to aggregate.

The concept of autophagy failure as a mechanism predisposing to cell death is relevant to pathogenesis in a range of diseases. Autophagy is necessary for the clearance of aggregate-prone proteins that are toxic, especially for post-mitotic cells like neurons [29]. Neuronal autophagy was initially believed to be relatively inactive, however, recent genetic studies using mice have highlighted the importance of constitutive autophagy in nondividing cells such as neurons [30,31]. Mice deficient for Atg5 or Atg7 specifically in neural cells develop progressive deficits in motor function that are accompanied by an accumulation of cytoplasmic inclusion bodies in neurons. These results demonstrate that constitutive autophagy is relatively active in neurons and that clearance of diffuse cytosolic proteins through basal autophagy is important for preventing the accumulation of abnormal proteins which may disrupt neural function, even in the absence of disease-associated mutations [30,31]. The compromise of autophagy as a possible contributor to different neurological diseases will be reviewed below with some examples.

### 4.1 Dynein

Eukaryotic cells transport molecules, complexes and organelles around the cell by means of energy-dependent motor proteins. The main motor responsible for movement of cargos to the minus end of microtubules is cytoplasmic dynein, which moves cargo centripetally towards the MTOC near the nucleus. Plus-end-directed kinesins move cargo in the opposite direction, centrifugally, outwards into the cytoplasm and the plus end of microtubules [32].

In contrast to most eukaryotic cells, neurons possess long highly branched processes, which make them exceptionally sensitive to defects in dynein function. Dynein and dynactin have multiple cellular house-keeping roles which include retrograde axonal transport, neurotrophic factor signalling, neurofilament transport, mRNA localization, neuronal migration, and protein recycling and degradation [32,33]. Disruption of dynein or dynactin is therefore expected to severely compromise the function and health of neurons. Mutations in the core of this motor – the dynein heavy chain – contribute to the pathogenesis of multiple neurodegenerative diseases [33,34] as well as produce motor neuron degeneration similar to what is seen in amyotrophic lateral sclerosis (ALS) [35]. Studies both in ALS patients and in transgenic animals have revealed decreased axonal transport in both anterograde and retrograde directions [36].

One key role for dyneins is to mediate the movement of autophagosomes along microtubules towards lysosomes clustered at the MTOC. This brings autophagosomes close to lysosomes, a prerequisite for subsequent fusion. The equilibrium that exists between autophagosome formation and clearance by lysosomes, termed autophagic flux, is microtubule-dependent, as the administration of microtubule-depolymerizing compounds such as vinblastine [37] or nocodazole [38] disrupts microtubule assembly and autophagosome transport to lysosomes, resulting in the rapid accumulation of autophagosomes, while preventing the turnover of LC3-II associated within these compartments. Under these conditions, autophagosome maturation and autophagosome–lysosome fusion are decreased, as autophagosomes are unable to shuttle from the cell periphery to the MTOC [11]. Indeed a similar increase in autophagosome number and LC3-II levels along with decreased autophagic flux can be observed in dynein-defective transgenic cells and mice [34].

### 4.2 Lysosomal storage diseases

Lysosomal storage diseases (LSDs) are a group of over 60 genetic conditions [39], most of which are caused by deficiency of lysosomal hydrolases leading to the accumulation of the corresponding substrate inside lysosomes. This, in turn, leads to a progressive accumulation of poly-ubiquitinated protein aggregates and of dysfunctional mitochondria [40]. A common cellular pathological feature in these diseases and their animal models is the manifestation of aberrant autophagic activity as evidenced by the accumulation of autophagosomes and increased levels of LC3-II, accompanied by neurodegeneration [41]. Deletion of genes encoding lysosomal enzymes such as cathepsins B and L in mouse brain [42], or pharmacological inhibition of lysosomal cysteine proteases [43] yield similar neurodegenerative states.

The first evidence for an involvement of autophagy in lysosomal storage diseases was obtained in a mouse model of Danon disease [44], in which the accumulation of autophagic vacuoles was observed in several tissues. Similarly, autophagosome accumulation was observed in neurons from murine models of neuronal ceroid-lipofuscinoses (NCLs)[45], as well as Pompe disease [46] in which a significant disturbance of the autophagic pathway was demonstrated.

A recent study [40,47] identified an inhibition block of autophagy in multiple sulfatase deficiency (MSD) and mucopolysaccharidosis type IIIA. MSD, an aggressive neurodegenerative disorder that results in death, is caused by mutations in sulfatase modifying factor (SUMF), the gene that encodes the formylglycine-generating enzyme (FGE) [48,49], required for posttranslational activation of sulfatases. Without this modification, sulfatase activity is impaired and the enzymes are therefore unable to degrade lysosomal contents. Analysis of the cell lines derived from MSD mice and their wild-type littermates show a decrease in the colocalization of the lysosomal marker, LAMP-1, and LC3 in MSD mouse embryonic fibroblasts (MEFs) [40,47], and the accumulation of autophagosomes resulting from impaired fusion of autophagosomes with lysosomes. This leads to a general impairment of autophagic protein degradation in these diseases.

### 4.3 The ESCRT complex

One of the important pathways involved in dendritic maintenance is the endosomal–lysosomal pathway, which plays a major role in the homeostatic regulation of transmembrane proteins. Endocytic cargos in early endosomes are either returned to the cell surface or trafficked to lysosomes for degradation. A critical step in degradation is the formation of multivesicular bodies (MVBs)—late endosomal compartments formed through the inward invagination and budding of vesicles into the lumen of endosomes. The fidelity of this process is maintained by the sequential interaction of four complexes ESCRT-0, -I, -II and -III plus several accessory components termed the endosomal sorting complexes required for transport (ESCRT) [12]. The specific interactions between these complexes are necessary for the formation of the MVB and proper progression of endosomal–lysosomal fusion.

In addition to roles in viral budding, cytokinesis, cancer and bacterial infection [50], the ESCRT complex is strongly implicated in neurodegenerative diseases [51]. The most direct evidence comes from the identification of individuals with missense mutations in the ESCRT-III subunit Vps2B who develop neurodegenerative diseases, like ALS and frontotemporal dementia (FTD), both characterized by progressive neuronal accumulation of ubiquitin-positive protein aggregates. Depletion of ESCRT-III or overexpression of Vps2b mutant proteins in cortical neurons presents with a similar phenotype [52].

Genetic and ultrastructural analysis in *Drosophila melanogaster* reveal that ESCRT-I, -II and -III, as well as their regulatory ATPase Vps4, are all essential for normal autophagy function [51,53]. Deficient ESCRT function results in elevated numbers of autophagosomes due to impaired autolysosome formation, and this is associated with the formation of large protein aggregates that contain the autophagy substrate p62 [12,54]. While some protein aggregates are seen within autophagosomes, large aggregates that are not membrane bound are also a prominent feature of this deficiency. The observation that autophagosomes contain decreased LAMP-1 in ESCRT-III-deficient neurons and in Drosophila models deficient for ESCRT-I and ESCRT-II, suggests that the normal ESCRT function is required for autophagosome–lysosome fusion [12,53].

### 4.4 Alzheimer disease

Alzheimer disease (AD) is a neurodegenerative disorder characterized by progressive dementia and brain morphological changes such as atrophy, senile plaques with fibrillogenic beta amyloid (Aβ), and intraneuronal neurofibrillary tangles (NFT) with hyperphosphorylated tau. In AD brain, Aβ accumulates within the large pool of autophagic vacuoles in swollen dystrophic neurites, suggesting that the autophagy system is involved in AD pathogenesis [55]. One probable contributor to autophagy deficiency in AD appears to be Beclin 1, whose expression is strongly reduced in the brains of AD patients to levels that would be predicted to impair autophagosome synthesis [56]. Genetic manipulations that decrease Beclin 1 levels in AD transgenic mice reduce neuronal autophagy, disrupt lysosomes, promote intracellular and extracellular Aβ accumulation, and enhance neurodegeneration. Conversely, increasing Beclin 1 expression results in diminished amyloid pathology in these AD transgenic mice [57]. These data raise the possibility that upregulation of autophagy may be beneficial in AD by decreasing the levels of Aβ that characterize its pathology. It is interesting that the prediction of these Beclin 1 data would be decreased numbers of autophagosomes in AD brains, yet the converse is seen in many cases with established disease [55]. One possibility to reconcile this apparent discrepancy may be that autophagosome synthesis is partially defective very early on, possibly before overt pathology appears, and that the Beclin 1 deficiency may play an important role in the genesis of disease. Then, as disease progresses, there may be additional events that impair autophagosome clearance, leading to a situation where, despite reduced autophagosome synthesis, there is a build up of autophagosomes due to a “traffic jam” in their removal processes. This may compound the autophagy problem and further reduce autophagic flux. Furthermore, the abnormal pool of slowly turned over autophagic vesicles may additionally impact on AD pathology by serving as a site for beta-amyloid generation [55].

### 4.5 Parkinson disease

In Parkinson disease (PD), death of dopaminergic neurons in the substantia nigra is associated with accumulation of α-synuclein within inclusions called Lewy bodies. Autophagy has been previously associated with PD through the protein PINK1, which is mutated in autosomal recessive forms of PD [58]. Full-length PINK1 interacts with Beclin 1 and this appears to act as a positive mediator of autophagy. A mutant form of PINK1 (W437X) does not interact normally and also lacks the ability to enhance autophagy, whereas these defects are not observed with another PINK1 mutant with impaired kinase activity [58]. Parkin, another protein that is mutated in autosomal recessive forms of PD, appears to act as a signal for selective autophagy of dysfunctional mitochondria [59].

## 5 Role of autophagy in clearance of aggregate-prone proteins—implications for therapeutic application

The two major routes for clearance of intracytoplasmic aggregate-prone proteins are the ubiquitin–proteasome system and the autophagy–lysosomal pathway. Whereas most large aggregate-prone proteins are precluded from entering the narrow pore of the proteasome barrel once they oligomerize, such proteins can be cleared by autophagy. The aggregate-prone species of such proteins (e.g., mutant huntingtin) are highly dependent on autophagy for their clearance, in contrast to the wild-type species [60,61]. Wild-type α-synuclein clearance can also be enhanced by autophagy upregulation [62].

### 5.1 Possible candidates for pharmacological induction of autophagy

#### 5.1.1 mTOR-dependent pathway

Chemical induction of autophagy protects cells against the toxic insults of aggregate-prone proteins associated with neurodegeneration by promoting their clearance. The very first known drug identified as an autophagy inducer is rapamycin, which was already in clinical use for other indications. Rapamycin is a lipophilic macrolide antibiotic originally used as an immunosuppressant. In mammalian cells, rapamycin inhibits the kinase activity of mTOR by forming a complex with the immunophilin FK506-binding protein of 12 kDa (FKBP12) [63,64]. Rapamycin acts specifically on the mTORC1 complex that suppresses autophagy when active.

Our studies have established that rapamycin treatment enhances the clearance of mutant huntingtin fragments, reduces aggregate formation and protects against toxicity in cell, Drosophila and mouse models of HD [60,65,66]. We also show that rapamycin promotes the clearance of a wide range of aggregate-prone proteins with polyglutamine- or polyalanine-expansion (including mutant proteins associated with spinocerebellar ataxias, mutant forms of α-synuclein implicated in PD, and mutant tau responsible for FTD), thereby attenuating their toxicity [62,67]. CCI-779, a rapamycin ester (i.e., an analogue of rapamycin) reduces both mutant huntingtin and ataxin-3 levels, thereby attenuating toxicity in mouse models of HD and SCA3, respectively [68,69].

In Drosophila, the beneficial effects of rapamycin against such proteins are autophagy dependent—no improvement is seen when autophagy is compromised [66,70,71]. These findings support the view that the primary benefits of this drug are mediated by autophagy and not by alternative mechanisms, like disruption of the translational machinery. Recently, delivery of the Beclin 1 gene was shown to induce autophagy and reduce amyloid and α-synuclein pathology in mouse models of AD and Parkinson/Lewy Body diseases, respectively [56,57]. This provides proof of principle for autophagy induction as a protective strategy in a wide range of neurodegenerative diseases.

In addition, autophagy induction confers additional cytoprotective effects by virtue of its apparent anti-apoptotic mechanism [72]. Rapamycin treatment and autophagy upregulation also protects cells against subsequent pro-apoptotic insults that are independent of aggregates (e.g., Bax overexpression and staurosporine in cell culture and paraquat toxicity in Drosophila [72]). Hence, upregulating autophagy has two distinct beneficial effects in neurodegenerative diseases: it promotes clearance of aggregate-prone proteins, as well as protecting cells against pro-apoptotic insults.

#### 5.1.2 mTOR-independent pathway

Although rapamycin is designed for long-term use, it has side effects which may make it unattractive to patients who may need to take the drug for decades. As far as we are aware, these side effects are unrelated to its autophagy-inducing properties. Thus, we and others have embarked on a series of studies to identify novel autophagy-upregulating compounds and have discovered pathways that are independent of the target of rapamycin. Inositol-lowering drugs, including mood-stabilizing drugs such as lithium, valproate and carbamazepine, can induce autophagy by inhibiting inositol monophosphatase (IMPase), leading to depletion of intracellular inositol levels and inhibition of the phospho-inositol cycle, as described above [23,73]. Indeed, such drugs enhance mutant huntingtin clearance and protect against its toxicity in cell and in vivo models [23,24,74], and thus may be potential therapeutic candidates for neurodegenerative diseases via autophagic clearance.

Another compound, trehalose, inhibits aggregation of mutant huntingtin and reduces toxicity in cells, and alleviates disease pathology in an HD mouse model [75]. Trehalose is a non-reducing disaccharide found in various non-mammalian species that protects cells against many environmental stresses, as it functions as a “chemical chaperone” that assists in protein-folding. We have recently identified trehalose as a novel autophagy inducer which promotes the clearance of aggregate-prone proteins like mutant huntingtin, and α-synuclein mutants (A30P and A53T) via an mTOR-independent pathway [75]. Besides, it also confers protection against cell death. In view of the diverse protective effects of trehalose in different models of proteinopathies, its prospect for treatment of neurodegenerative diseases warrants further consideration for development into clinical use.

### 5.2 Screening of compounds—SMERs and various drugs

To explore other novel therapeutic agents capable of modulating autophagy for the purpose of treating neurodegeneration, high-throughput chemical screens were performed and various small molecule inhibitors (SMIRs) and enhancers (SMERs) of the growth-suppressing properties of rapamycin in yeast were identified [76]. Among these, three SMERs (SMERs 10, 18 and 28) were subsequently confirmed to induce mTOR-independent autophagy in mammalian cells as they increased clearance of autophagy substrates such as A53T α-synuclein and mutant huntingtin and reduced huntingtin toxicity in the Drosophila HD model [76].

In addition, further screening of a library of FDA-approved drugs/compounds was carried out in order to search for alternative therapeutic candidates to rapamycin. This screen revealed that L-type Ca^2+^ channel antagonists (e.g., verapamil, loperamide and amiodarone), the ${\text{K}}_{\text{ATP}}^{+}$ channel opener minoxidil and the Gi-signalling activator clonidine [24] induced autophagy. These compounds induce autophagy by acting on the cAMP–Epac–PLCɛ–IP_3_ pathway, which modulates calpain activity by influencing Ca^2+^ levels, as described above. It is notable that the drugs acting on this pathway enhance mutant huntingtin clearance via an mTOR-independent autophagy pathway, decrease mutant huntingtin aggregate levels and confer protection against toxicity in HD cell, fly and zebrafish models [24,77].

## 6 Future perspective

The prospect of upregulating autophagy as therapy for certain neurodegenerative diseases (e.g., Huntington disease) nonetheless raises some challenges. In principle, earlier treatment would be desired to delay the onset of disease. As most patients will have family history, it is possible to identify at-risk individuals by genetic screening [78]. Although rapamycin has been tailored for long-term use [79], it still has side effects due to inhibition of mTOR, which regulates many other cellular processes independent of autophagy. One consideration is that this approach may not require continuous autophagy upregulation. Indeed, the “pulsatile” induction of autophagy with spaced rapamycin administration was the strategy we employed in our mouse studies [72,80], and this may be applicable in humans with possibly even longer gaps between doses. However, other alternatives such mTOR-independent drugs or a combination of mTOR-dependent and -independent therapy may be more suitable for long-term use. As such, a detailed understanding of pathways regulating autophagy will be of paramount importance in the context of neurodegenerative disease.
